# Health economic evidence in clinical guidelines in South Africa: a mixed-methods study

**DOI:** 10.1186/s12913-021-06747-z

**Published:** 2021-07-26

**Authors:** Maryke Wilkinson, Karen J. Hofman, Taryn Young, Bey-Marrié Schmidt, Tamara Kredo

**Affiliations:** 1 Cochrane South Africa, South African Medical Research Council, Tygerberg Cape Town, South Africa; 2 SAMRC Centre for Health Economics and Decision Science, PRICELESS SA, School of Public Health, Faculty of Health Sciences, University of the Witwatersrand, Johannesburg, South Africa; 3 Centre for Evidence-based Health Care, Division of Epidemiology and Biostatistics, Department of Global Health, Faculty of Medicine and Health Sciences, Stellenbosch University, Tygerberg Cape Town, South Africa; 4 School of Public Health, University of the Western Cape, Bellville Cape Town, South Africa; 5 Division of Clinical Pharmacology, Department of Medicine, Faculty of Medicine and Health Sciences, Stellenbosch University, Tygerberg Cape Town, South Africa

**Keywords:** Economic evidence, Evidence-informed decision-making, Survey, Focus group, Clinical practice guidelines

## Abstract

**Background:**

Evidence-informed clinical practice guidelines (CPGs) are useful tools to inform transparent healthcare decision-making. Consideration of health economic evidence (HEE) during CPG development in a structured manner remains a challenge globally and locally. This study explored the views, current practice, training needs and challenges faced by CPG developers in the production and use of HEE for CPGs in South Africa.

**Methods:**

This mixed-methods study comprised an online survey and a focus group discussion. The survey was piloted and subsequently sent to CPG role players - evidence reviewers, CPG panellists, academics involved with training in relevant disciplines like health economics and public health, implementers and funders. The focus group participants hold strategic roles in CPG development and health economic activities nationally. The survey evaluated mean values, measures of variability, and percentages for Likert scales, while narrative components were thematically analysed. Focus group data were manually coded, thematically analysed and verified.

**Results:**

The survey (*n* = 55 respondents to 245 surveys distributed) and one focus group (n = 5 participants from 10 people invited) occurred between October 2018 and February 2019. We found the most consistent reason why HEE should inform CPG decisions was ‘making more efficient use of limited financial resources’. This was explained by numerous context and methodological barriers. Focus groups participants noted that consideration of complex HEE are not achievable without bolstering skills in applying evidence-based medicine principles. Further concerns include lack of clarity of standard methods; inequitable and opaque topic selection across private and public sectors; inadequate skills of CPG panel members to use HEE; and the ability of health economists to communicate results in accessible ways. Overall, in the absence of clarity about process and methods, politics and interests may drive CPG decisions about which interventions to implement.

**Conclusions:**

HEE should ideally be considered in CPG decisions in South Africa. However, this will remain hampered until the CPG community agree on methods and processes for using HEE in CPGs. Focused investment by national government to address the challenges identified by the study is imperative for a better return on investment as National Health Insurance moves forward.

**Supplementary Information:**

The online version contains supplementary material available at 10.1186/s12913-021-06747-z.

## Background

Healthcare funders, commissioners and operational managers in the public and private sectors are expected to select, fund and implement health interventions and services that represent the best use of available health resources in their local context. Clinical practice guidelines (CPGs) that are informed by systematic reviews of the available clinical and economic evidence are useful tools to aid transparent healthcare decision-making [1, 2]. Health economic evidence (HEE) can be produced using a variety of methodological approaches such as budget impact assessment, cost-effectiveness analysis or cost-utility analysis to name a few. The type of HEE produced and the degree to which it is incorporated in CPG development depend on the intervention type, data availability, and the availability of technical skills of those synthesising the evidence and those who need to use the evidence. Ultimately, the inclusion of the best available effectiveness and economic evidence should enable the decision-maker to make a more informed decision.

In South Africa, the Essential Drugs Programme at the National Department of Health supports the creation and maintenance of the national Essential Medicines List (EML) by coordinating the production of systematic reviews and costing analyses, including Health Technology Assessments (HTAs), and convening ministerial-appointed committees to appraise evidence and make decisions regarding the selection/deselection of technologies to the EML. Essential Medicines List decisions feed into the production of the Standard Treatment Guidelines, which are the implementation mechanisms for the EML and provide guidance to health care professionals on the rational use of the essential medicines at a particular level of care. Currently the scope of the HTAs and analyses that inform EML decisions vary depending on the review question, with economic analyses only conducted and reported for selected topics. The clinical and economic evidence informing private sector formularies, and by extension treatment protocols, are also variable, with HTA processes and evidence requirements differing significantly between organisations.

Recent research on CPG development and practices by the South African Guidelines Excellence (SAGE) project highlighted the need to improve CPG quality, training, development and reporting methodology, and to increase the involvement of end-users in the CPG development process [3]. Internationally, published literature and governmental publications indicate the need for more rigorous methods and processes to ensure CPG recommendations are informed by the best available evidence, including cost-effectiveness, resource use and equity [4–6]. It also shows that the utilisation of HEE in the production of CPGs by health authorities is already ongoing to varying degrees [4, 7–9]. For example, HEE is taken into account when making medicine reimbursement decisions globally, including in South Africa [10], and in some cases these decisions are reflected in related CPGs. However, the manner in which economic evidence is used in CPG development is less explicit [7], less agreed upon or understood [11], and still very much an evolving discipline [12]. This might partly be due to the fact that the type of HEE and the manner in which it can be used in CPG development is dependent on country specific health systems, benefit packages, budgets, and value preferences [4, 7, 11, 12]. Therefore, even though there are basic underlying principles of how HEE can be used to inform CPG recommendations, the detailed processes and methods used in a specific country or health system should be based on its particular needs, constraints and value preferences.

The South African healthcare sector operates in a constrained resource environment with a complex, two-tier system. The private health insurance sector covers approximately 16% of the population and the remaining population is covered by the public sector with funding from both national and provincial sources (mostly provincial). This situation leads to substantial differences in healthcare costs, demands, financing mechanisms, benefit packages and incentives [13, 14]. The private healthcare market was recently investigated by the Competition Commission (Health Market Inquiry) with findings and recommendations published in 2019 [13]. The Inquiry found that the private health care market is characterised by “high and rising costs of healthcare and medical scheme cover, and significant overutilization without stakeholders having been able to demonstrate associated improvements in health outcomes” [13]. Among the issues identified is a lack of transparent, standardised methods to measure and compare the cost-effectiveness of healthcare interventions [13].

Through a financing mechanism called National Health Insurance (NHI), South Africa has introduced and is planning a series of reforms over the next few decades to introduce Universal Health Coverage (UHC) [15]. The intention is to improve the quality of care in the public sector and to better co-ordinate health provision across the public and private sectors. The health benefits that will be provided will require development of CPGs based on the best available clinical and cost-effectiveness evidence [15]. However, CPG development is currently fragmented, with no formal co-ordination between the public and private sector in terms of topic selection, methodological approaches, reporting standards or implementation strategies [16].

Cochrane South Africa and the Centre for Evidence Based Health Care (both SAGE partners), and the South African Medical Research Council Centre for Health Economics and Decision Science, PRICELESS SA, initiated this study to understand if and how HEE could be used to inform CPG development in South Africa. As a starting point, the research team considered it essential to identify role players and better understand the attitudes, technical experience and needs, and challenges faced by CPG developers in relation to the production and use of HEE in CPGs.

## Methods

The aim of the study was to assess the views, current practice, training needs and challenges faced by CPG stakeholders in relation to production and use of HEE in CPG development. The specific objectives were to identify the views, including barriers and facilitators, by CPG stakeholders involved in the production and use of HEE in CPG development; determine the extent to which HEE are currently produced for and used in CPGs; and explore the technical capacity and training needs of CPG stakeholders with respect to production and use of HEE.

### Design of the study

This mixed-methods study consisted of an online survey followed by a qualitative focus group discussion [17]. By combining qualitative and quantitative methods we were able to deepen the understanding of data through triangulation of results collected from different sources [18].

Survey: An online survey (in the form of a structured questionnaire) aimed to collect descriptive qualitative and quantitative data from a predefined group of respondents. Respondents completed the online survey independent from a researcher and at their own convenience during a specified time period [19].

Focus group: The focus group discussion aimed to verify survey results as well as identify areas not adequately addressed by the survey and prioritised content for further HEE capacity building. Focus group participants were encouraged to communicate with one another, exchange ideas and comment on each other’s experiences or points of view. This approach was useful because participants reacted to and built on the responses of others, and it allowed the facilitator to probe participants to elaborate on specific issues or ask them for clarification [19].

### Eligibility and sample

Survey: Targeted invitations were emailed to CPG developers (purposive sampling method). The term ‘CPG developers’ comprised all role players involved CPG development, including producers/ synthesisers of evidence; members of CPG panels; institutions that provide training in relevant disciplines such as health economics, biostatistics, clinical epidemiology, and public health; individuals involved in CPG implementation; and funders of CPG development and implementation. CPG developers were identified from a CPG mapping project [16], SAGE CPG interest group membership, an online search for South African stakeholders who published articles related to health economics in the last 10 years, and a review of institutions/departments providing analytical services or formal training in health economic evidence production. In addition, survey recipients were encouraged to share the survey with other stakeholders in similar roles or who could provide insight to the study area (snowball sampling method). There were no limits on the sample size to enable this survey to capture as broad an input as possible. The survey was sent to 245 people involved in CPG development, use or implementation in October 2018.

Focus group: Following the survey analysis, a focus group was held in February 2019. Participants were purposively selected, based on knowledge of stakeholders that play a strategic role in CPG development and/or HEE production and training activities. Ten stakeholders were invited to participate in the focus group discussion and five were available to participate on the day of the focus group due to competing work commitments.

### Description and use of study materials

Survey: No validated questionnaires addressing all the research objectives were available. For this reason, a new questionnaire was developed with two questions adapted from a survey produced by the Health Intervention and Technology Assessment Program (HITAP) in Thailand [20]. This was included to address objective one about *preferences and challenges of CPG developers in relation to production and use of HEE in CPG development*. Additional questions were formulated to address other study objectives based on key considerations and concerns regarding the use of HEE in CPGs from published literature [4, 7, 12, 21] and suggestions by South African experts in the field of health economics and CPGs. The survey adhered to the STROBE checklist for cross-sectional studies [22]. English was an acceptable language for the study population. The questionnaire was piloted with a representative sample (*n* = 9 respondents) to ensure the questions were easily understandable and addressed the study objectives. Survey questions were revised based on this feedback before sending it to the survey sample population. Sixty responses (out of 245 surveys distributed) were received of which 55 participants were eligible. Participants typically spent 26 min completing the survey and the average completion rate was 73%. All survey respondents answered a qualifier question to ensure they were eligible to participate (respondents were asked to select or state their CPG-related experience). The survey was administered electronically, using an online survey tool (SurveyMonkey®). A reminder was sent one week before the survey closing date. Using the functionality of SurveyMonkey® tool, the ‘Anonymous Responses’ option was selected, therefore no personally identifiable data were collected about the survey participants. All eligible participants gave their consent to participate and agreed that their anonymised survey response could be used in future research reports or publications.

Focus group: Specific questions were prepared in a semi-structured topic guide. Questions were based on the survey findings and gaps in this data. The focus group was held face-to-face at the School of Public Health at the University of Witwatersrand in Johannesburg, and conducted by an experienced facilitator. Key findings from the survey were presented to the focus group participants, after which they were encouraged to ask questions and provide feedback, thereby verifying or refuting the results. The focus group discussion was digitally recorded with the permission of the participants, and the audio-recording was transcribed verbatim and checked against the recording for accuracy.

### Analysis

Survey data were analysed using quantitative and qualitative methods. Summary methods were used to evaluate mean values and measures of variability, and percentages to evaluate the Likert scales. Narrative components were thematically analysed.

Focus group data were manually coded independently by two researchers (MW, BMS) and a third researcher checked the codes (TK) [19]. These were then presented to the entire research team in an analysis meeting for verification and agreement. A deductive approach was used, in which the survey questions were used as the key themes; together with an inductive approach in which emergent themes addressing general issues relating to the use of HEE in CPGs were captured.

## Results

We report the results of a survey and the subsequent focus group discussion. Fifty-five eligible participants responded to 245 surveys distributed. The respondents had a variety of CPG-related experience ranging from evidence production (*n* = 32); evidence use (*n* = 31), with fewer involved with training (*n* = 20); implementation (*n* = 19) and/or funding (*n* = 8). Most were aged 36–50 years (44%, 24/55), followed by those over 50 (38%, 21/55) and those aged 18–35 years (18%, 10/55). The majority (93%) were from the Western Cape Province (42%, 23/55), Gauteng (42%, 23/55) and Kwa-Zulu Natal (9%, 5/55). Many respondents worked in academic or research environments (45%, 25/55) and almost 80% (43/55) held a masters (30/55) or doctorate (13/55) qualification.

The five focus group participants were from Gauteng Province (*n* = 2), Eastern Cape (*n* = 1) and Western Cape (n = 2) and each had multiple roles relevant to this study, namely: evidence producers; evidence users; funders; training or research institutions; or implementation specialists. They represented academia, a professional association (private sector), and the National Department of Health, and by discipline represented healthcare providers (medical specialist, pharmacist), public health specialists, and health economists.

### Views on production and use of HEE in CPG development

**Fig. 1:**
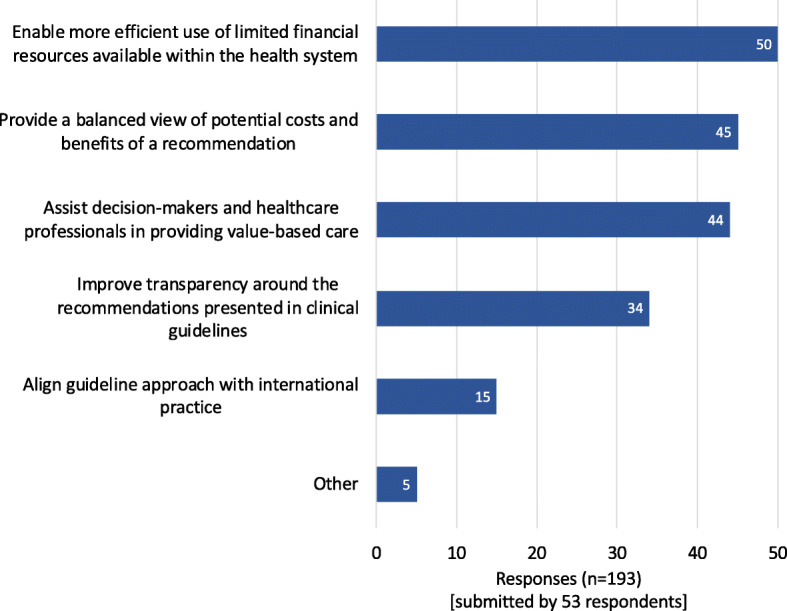
Reasons that economic evidence should be considered in CPG development

Most survey respondents (48/53) considered the use of HEE in CPGs very important (30/53) or quite important (18/53) and selected key justifications of why HEE should be used in CPG development (Fig. 1). ‘Making more efficient use of limited financial resources’ was considered the most important, followed by the consideration that HEE allows a more ‘balanced view of the potential costs and benefits of a CPG recommendation’. The importance of the affordability and sustainability of CPG recommendations were highlighted by some respondents under the ‘other’ option. Focus group participants echoed these justifications with one participant stating that *‘if the final decision-making body in this country is not using health economic information; we’re in a really horrible space’* [FGP_5].

### Reasons that HEE is not used in CPG development

**Fig. 2:**
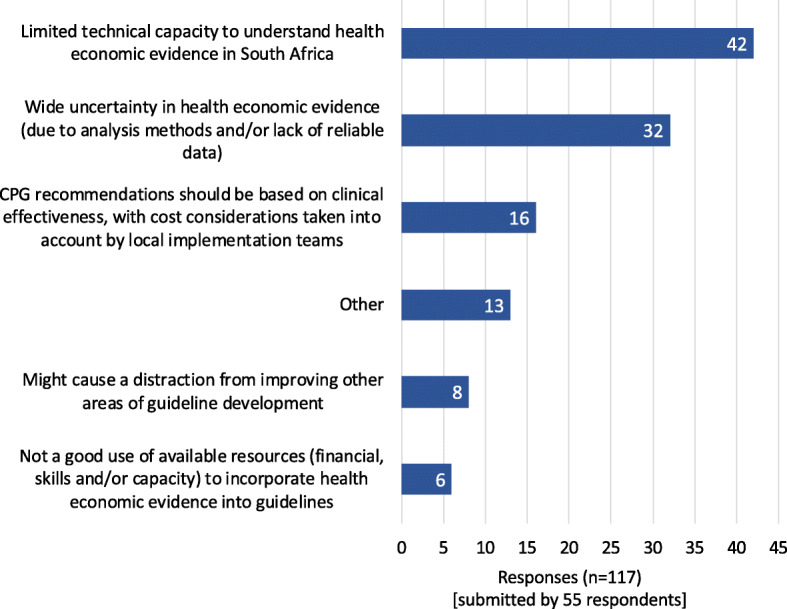
Reasons for not including economic evidence in guideline development

‘Limited technical capacity’ was cited as the most important barrier to the use of HEE in CPGs (42/55 respondents), followed by ‘the wide uncertainty in the results of economic evaluations due to analysis methods and/or lack of reliable data’ (32/55 respondents) (Fig. 2). Additional barriers to the use of HEE in CPGs were provided under the ‘other’ category, and included limited understanding of the role of HEE (and its underlying principles); weak leadership in raising awareness of this role; poorly managed conflicts of interest; limited funding and skills; and non-transparent topic selection and scoping practices for economic evaluations. Focus group participants expressed concern that there is a lack of clarity about which kinds of HEE analyses are required for particular situations. This is compounded by focus group participants’ experience that incorporating HEE in decision-making adds complexity to an already difficult process. Some focus group participants felt that CPG developers already struggle with application of evidence-based medicine principles and so adding further requirements may be ‘*pointless*’ until guideline methods are better implemented.

### Topic selection for HEE analyses

**Fig. 3:**
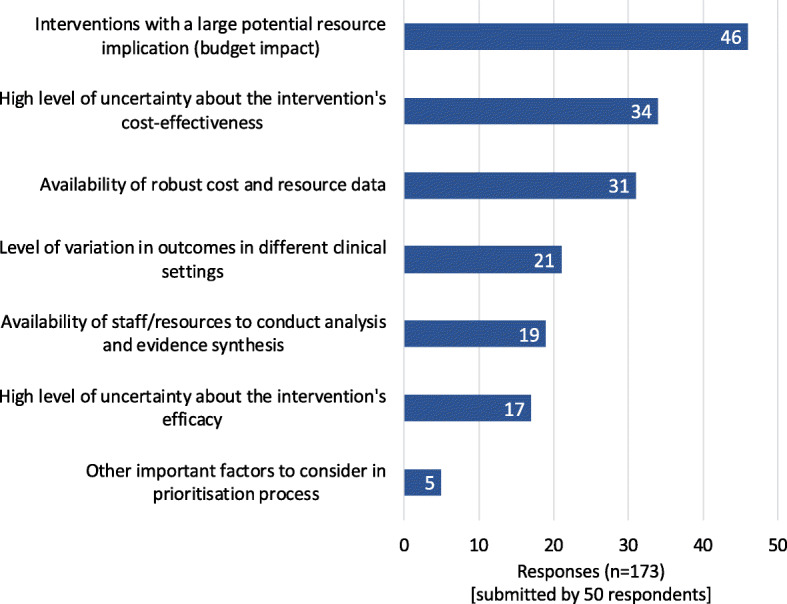
Factors to consider in selection of topics for health economic evaluation

The potential resource implication of introducing an intervention was considered the most important factor for topic selection (46/50 respondents), followed by high-level uncertainty about cost-effectiveness (34/50) and the availability of robust data (31/50) (Fig. 3). Additional topic selection criteria included making better use of and adapting internationally produced evidence when possible so that available resources can be more efficiently used, responding to stakeholder requests, and the wider economic and clinical impact of an intervention. Focus group participants proposed that South African CPG developers should be ‘*pragmatic*’ and that ‘*not everything needs to be subjected to economic evaluation. Sometimes we will do the back of the matchbox calculations, and that is sufficient*’. Full economic evaluations may be needed for *‘very costly’* or *‘new interventions’* or when benefit is considered *‘borderline’*. Focus group participants thought that topic selection was impacted by the limited capacity and resources available to invest in conducting economic analysis, resulting in other drivers for topic selection, as described in these quotes: ‘*decisions are not made on evidence, they are made on political whim*’ [FGP_2] and that many ‘*ground decisions are purely politics or personalities*’ [FGP_5].

### Methods-specific issues affecting use of HEE in CPGs

Most respondents (49%, 25/51) were unsure if the methods currently used to gather, analyse and report economic evidence could usefully inform CPG development, while 29% (15/51) said the methods are useful and 22% (11/51) said they were not. Forty-four respondents provided specific methodological issues they considered a barrier to the use of HEE in CPGs such as availability of clinical and costing data; lack of standardisation; poor reporting of results; lack of transparency; lack of skills and funding; and issues relating to the disparity between private and public (state) funded care. Proposed solutions included improved reporting standards; investment in clinical and costing research; training; and the use of standardised methodology. A thematic analysis to understand methodological issues and proposed solutions was conducted and is presented in Table 1. Focus group participants agreed with the survey respondents regarding differing methodologies and reporting and lack of agreement on standards perceived as ‘*a problem globally not just for a particular country’* made more fraught by the politics and the *‘contested space and … .. varied views and varied lobby groups who want to have a slice of that healthcare budget’* [FGP_5].

**Table 1:** Methodological barriers to health economic evidence use in CPG development

Thematic areas	Sub-themes	Solutions
1. **Lack of agreed methods and reporting standards**	• Inconsistent methods / poor standardisation (e.g. clinical end-points or comparators selected) • Poor reporting of methods • Methods not agreed by stakeholders in South Africa	• Develop methods and reporting standards informed by broad based stakeholder consultation
2. **Lack of available data**	• Data from South Africa of costs: - Costs vary by sectors (i.e. public vs. private), settings (e.g. provinces) and levels of care (i.e. primary vs secondary) - Poor availability of costing data • Outcomes data to inform decisions	• Strengthen data collection systems • Develop registries for data collection • Investment in research
3. **Lack of skills to conduct analyses and use economic evidence**	• Lack of trained health economists, statisticians, mathematicians, team with multi-disciplinary skills • Lack of skills for CPG developers to read and use HEE	• Train economists and statisticians to work on HEE for CPGs
4. **Lack of funding/ insufficient resources**	• Training and building necessary skills • Collecting data/evidence to inform economic models (e.g. clinical outcomes, registries)	• Investment in research and training
5. **Lack of trust and inability to share**	• Poor sharing of available data (e.g. private to public)	• Develop agreements between groups to share data respectfully

### Context-specific issues affecting use of HEE in CPGs

**Fig. 4:**
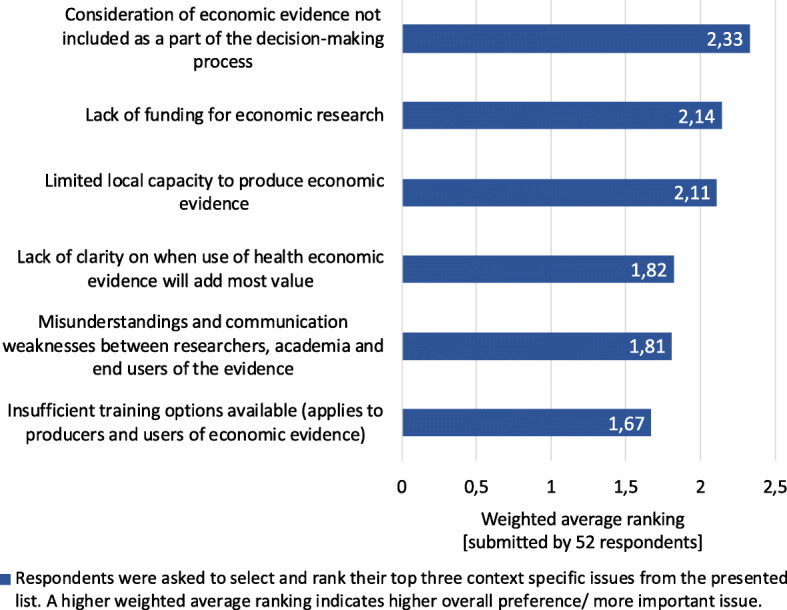
Ranking context-specific issues that may affect health economic evidence use in CPGs

Context-specific issues are challenges to the processes or environment under which a CPG is produced, and therefore not easily changed with adjustments to the methodological or reporting specifications [20]. Context-specific issues posed a greater barrier to using HEE in CPGs than the methods challenges. The lack of a formal process for consideration of HEE in CPG development and funding for economics research were considered the most important context-specific issues affecting use of HEE in CPGs in South Africa (Fig. 4). Potential solutions to the context-specific issues (suggested by survey respondents) are summarised in Table 2. Focus group participants agreed with the survey findings that HEE is not consistently included in decision-making processes. At the heart of this issue was that the demand side (what policymakers/ funders want or need) is poorly articulated. An academic colleague explained it, *‘I think the problem is one of the market. Until you have a market that is specifically saying: “yes we are going to use you, and this is what we want”, there is no actual consumer base here that is particularly powerful*’ [FGP_5]. It was also mentioned that the private and public sectors have different strategies and different capacity to pay high prices for HEE work, with the public sector ‘*always running along, panting behind because the state can’t afford it* [HEE]’ [FGP_5]. As the country shifts towards the national health insurance, it was corroborated that ‘buy-in’ from top level government departments including the Presidency and the Treasury is required to recognise the value of HEE to drive return on investment. This would enable informed decisions regarding new technologies as well as disinvestment in out-of-date technologies.

**Table 2:** Solutions to contextual challenges affecting the use of economic evidence in CPGs

Solutions for production and use of HEE	
• Need for political will and backing • Training of guideline panels, policymakers and health economists • Include health economists and others in expert review of analyses • Health economists to suggest guideline recommendations based on the health economic evidence and work to increase the transparency and credibility of analyses • Public-private partnerships to share evidence and for cross-sectoral decisions • Clarify outcomes that matter to decision-makers for use in economic evaluations • Need centralised management of health technology assessment (to commission and fund), a guiding body to coordinate functions	

### Current technical ability of CPG developers to enable the production and use of HEE in CPGs

Most respondents (more than 92%) considered it very or quite important that producers of HEE should be able to conduct all the listed types of analyses: Identification, gathering and synthesis of costing data; analysing and reporting costing data; budget impact analysis; and economic evaluations (including cost-minimisation analysis, cost-effectiveness analysis, cost-benefit analysis, and cost-utility analysis). In addition, they suggested that HEE producers should conduct simple cost comparisons, impact analyses, programme costing, optimisation analysis, multi-criteria decision analysis, and interpretation of external factors that might affect healthcare costs. The respondents themselves mostly had experience in commissioning, analysing or using ‘costing analysis’, followed by ‘budget impact analysis’ and ‘locally produced economic evaluations’. Respondents had limited experience in utilising ‘economic evaluations produced outside of South Africa’. In terms of their own experience in producing HEE, the respondents had the most experience in ‘identifying, gathering and synthesising costing information’, followed by ‘analysing and reporting costing data’, ‘budget impact analysis’ and ‘economic evaluation’. Almost half of the survey respondents (49%, 24/49) were aware of situations in which economic evidence was produced to inform South African CPG development however the majority (67%, 33/49) were not aware or were unsure if CPG developers in South Africa formally consider HEE when making recommendations. Focus group participants emphasised the limited capacity of CPG panel members to read, understand and use the HEE in their decision-making. Further, there are differences between the private and public sector regarding what is needed, used and how economic analyses are funded, which as one academic colleague suggested currently leads to a: ‘*fight between industry funded analyses, funder funded analyses and the state’* [FGP_5].

### Training needs of CPGs developers to produce and use HEE in CPGs

**Fig. 5:**
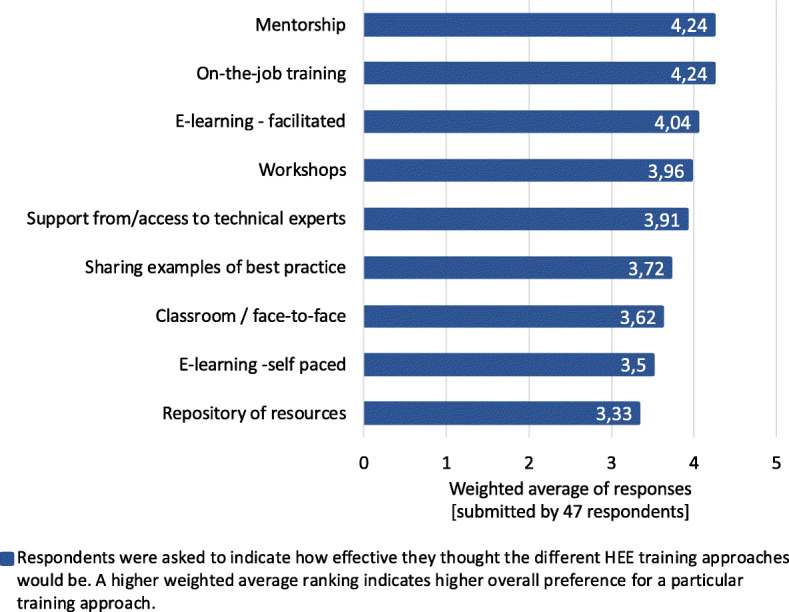
Effectiveness of different approaches to training in economic evaluation

The three areas in which respondents felt that the CPG producing community and they themselves would benefit from additional training was in identification, gathering and synthesis of costing information; structured decision-making process for incorporating HEE into CPGs; and producing and/or interpreting health economic analysis. They provided examples of relevant courses and considered various training mediums effective (Fig. 5), with mentorship and on-the-job training considered the most desirable overall. Focus group participants stressed that one first needs clarity regarding which analyses need doing and for whom before planning training. They also felt that health economists may be trained in the field but lack the ‘*real world’, ‘pragmatic’* capacity to undertake these economic analyses in responsive, accessible, timely and usable ways.

### Who needs to build skills, what skills do they need to build and how do we take it forward?

Focus group participants identified various role players who need training: research institutions, CPG funders, CPG panels, various forms of government, funders (including treasury), health professionals, clinicians, CPG end users, professional societies, industry, pharmaceutical industry, civil society. For CPG developers, the capacity to apply the principles of evidence-based medicine, and not only understand them, is important. They should have capacity to make decisions in the face of uncertainty in the research evidence; and they need to understand the principles and reports of HEE. Formal training for health economists was not thought to be an issue, but rather their ability for ‘*translating the complex stuff into common day language*’[FGP_2]. Thus, what was re-iterated was the need for ‘*on the job’* training and being embedded in the positions with clear career pathing so that those trained can have jobs in the public sector and not only in the private sector. In the contested space of making decisions where ‘*political decision-making trump [s] good economic analysis’*, health economists need skills to ensure that ‘*economic evaluation is packaged and coherent enough and inspiring enough for decision-makers to use’* [FGP_2].

Overall, participants made it clear that training is not the primary issue and can’t be planned until other more pressing issues are resolved, such as buy-in, political backing and clarity regarding what is needed on the ‘demand side’ to inform training. It was suggested that until you ‘*actually define how you are going to use that person [health economist], the whole debate about how they are going to act becomes redundant. You really need to find out what tools you actually need for decision-making in the country. And then work backwards from that to how you would change such people and get them to interact with the system, rather than generate another pie-in-the-sky guide on a perfect health economist*’ [FGP_5].

## Discussion

Survey and focus groups participants agreed that HEE should be used in South Africa to inform CPG decisions, particularly as the resource constrained context requires judicious spend on health. Notwithstanding this common agreement on the importance of economic considerations for decision-making, the methods, approaches and actual practice of using HEE in CPGs remain challenging. Globally, methods for using HEE in CPGs have advanced over the past few decades, with increasing numbers of ‘guideline for guidelines’ manuals outlining processes for their CPG group’s needs. Some of these methods are captured in the Guidelines 2.0 checklist which systematically considered all guideline manuals and created a listing of all key steps CPG groups should consider, including consideration of costs and resources in decisions [2]. Despite evolving methods for conducting economic analyses, for our participants, the major question was not how to do analyses (although that was part of it) but rather which types of economic analyses are required to inform specific decisions and how to ensure that HEE reporting could be provided in standardised, useful, and accessible ways. Planned reforms under NHI [15, 23] have the potential to strengthen and integrate South Africa’s HTA capacity and processes, which may result in improvement in the methods used to select, produce and report HEE. This could lead to locally produced HTAs being a more consistent and valuable source of HEE that can be used to inform national treatment guidelines and benefit packages. Health technology assessments, costing analyses and economic evaluations produced outside of South Africa are other important reference sources for HEE. Work facilitated by the GINAHTA working group (represented by Guidelines International Network and the International Network of Agencies for Health Technology Assessment) [24] may provide useful insight on how and when HTAs developed outside of South Africa can be best utilised in the local setting.

Participants, particularly focus group members, suggested that basic costs should generally be considered during CPG processes, with complex economic analyses reserved for new or expensive technologies. For the most part, it was suggested that ‘back of the envelope’ or less formal considerations of costs could suffice. This aligns with emerging guidance from multilateral organisations such as the World Health Organisation (WHO), national governments and professional societies who have also grappled with these challenges to clarify which economic analyses to do when to ensure transparent, equitable decision-making [25]. The feasibility of producing relevant and useful HEE for CPGs should also be considered - for example, it will be more difficult to produce HEE for topics that cover broad clinical areas compared to topics with a more narrow scope [26]. The American Thoracic Society (ATS) have developed guidance on using costing data to inform CPG decisions utilising pragmatic approaches to ensure complex analyses are only conducted when needed and likely to be used, and accessible data on acquisition costs and resources are presented more often [7]. Both WHO and ATS organisations have adopted rigorous CPG methods with use of systematic reviews and systematic decision processes, such as the use of Grading of Recommendations Assessment, Development and Evaluation (GRADE) evidence to decision tables [27]. As such, what sets these CPG developers apart from the South African CPG community is that these groups have established skills in evidence-based decision-making and have built on that capacity to further plan when and what to use for informing costs, resources and costs-effectiveness for CPG recommendations [7, 25]. Our focus group participants emphasised insufficient skills in evidence-based medicine in local CPG groups, adding that the addition of cost considerations, particularly more advanced costing analyses, adds to the complexity and might hinder rather than help decision-makers.

Participants identified multiple methodological and contextual obstacles explaining why HEE is currently not used to inform CPGs. In addition to the issue of complexity when adding HEE to decision-making, further issues were identified such as inadequate guidance about which HEE is needed for which decisions (the ‘market’); lack of local data and skills in using global data; and unclear or lack of standardised methods for economic analysis. Hence, despite evolution in methods guides globally outlining how and when to include economic analyses, our participants reported that there is insufficient methods guidance in South Africa. Further, in the absence of strong, accepted methods, financial interests are perceived to influence and drive decisions in CPGs.

Priority setting is a key activity in CPG development or technology assessment [2, 28]. This is highlighted in local processes for priority setting for topics for HEE which was perceived to be driven by financial or non-financial interests in the absence of methods for doing this transparently.

Overall, contextual issues emerged as more pressing than methodological limitations. A particular contextual issue was South Africa’s two-tiered public and private healthcare system which has been reported to result in inequitable access to quality health care and impacts CPG development processes and implementation [29, 30]. The fault lines between private and public sectors was thought to impact transparency and consistency across sectors resulting in inequitable access to health care services. Further views by focus group participants indicated that the private sector may have easier access to costly economic analyses which the public sector could not access or afford. A proposed solution from the survey was centralised oversight of guidelines and HEE set standards to ensure transparency - an approach that echoes suggestions from other South African CPG research in which national stakeholders identified gaps in oversight leading to opportunities for interests to drive decisions and priorities [29]. Models of centralised health policy decision-making oversight is seen in some settings as outlined in South African Guidelines Excellence (SAGE) report of all CPG agencies describing the role and functions [31]. Several examples exist, one that is particularly relevant to SA is the National Health Intervention and Technology Assessment Program (HITAP) in Thailand where methods for considering HEE are clearly outlined and evolved from the need to ensure transparency and build public trust [32, 33].

Training and technical capacity in conducting and using HEE for CPGs was explored. Focus group participants recommended that all groups involved with decision-making including researchers, economists, and policymakers need to build skills in HEE appraisal and use. It was emphasised in the focus group that for health economists, training provided through tertiary institutions is not the problem, and that courses likely include all necessary competencies. In certain academic institutions, the need for capacity strengthening has been recognised and capacity building in health economics is proceeding at both the masters and doctoral level. However, the issue is the gap in translating findings into policy especially when health economists present highly technical reports which users such as CPG developers do not have the capacity to decipher. They suggested that what is needed are applied skills, communication skills, and career opportunities (particularly in the public sector) to work and learn ‘on the job’.

Our participants were clear that HEE are not likely to inform policy until certain issues are addressed. Firstly, clarity from South African decision-makers is required on what types of HEE are needed for what decision, after which HEE can be produced using rigorous, standardised methods and reported using transparent reporting guidelines. Next, opportunities for career pathing, on the job training, and mentorship is needed – on the one hand to provide health economists with clarity on what reports are needed and how to translate them for CPG developers and policymakers. This would be needed in useful, accessible language. In addition, there is a need to build capacity amongst CPG developers to appraise and use HEE - to together work on bridging the gap between HEE reports and what is needed for a decision.

### Limitations and strengths

This study addressed the broad question of the current practice, training needs and challenges faced by those involved with CPGs and health economics in relation to production and use of HEE in CPG development. The survey, followed by a focus group, allowed broad engagement with multiple stakeholders with deeper exploration among those closely involved with CPG activities.

A potential limitation was whether the sample size was sufficient in terms numbers of people who are actively involved with CPG. In order to mitigate this, we used as broad a sampling approach as possible, including a snow-balling approach so that the survey could be shared further. The fact that the sample is skewed to participants from two provinces - Gauteng and the Western Cape - likely reflects the major training institutions and inequitable distribution of academics in South Africa who contribute to CPG development. Furthermore, the focus group size of five was smaller than the anticipated ten participants. Despite this, the major anticipated stakeholder groups were all represented and rich inputs were provided that reflected and clarified views from the survey process.

Strengths of the study include the fact that respondents spent on average 27 min completing the survey. One interpretation is that participants had an interest and commitment to contributing to this policy development.

## Conclusions

One of the earliest methods guides for developing CPGs stated: “health interventions are not free, people are not infinitely rich, and the budgets of [health care] programmes are limited.” [34–36]. Study respondents reinforced the view that affordability and consideration of financial resources should always be part of CPG decision-making.

As South Africa continues in its trajectory to UHC aiming for an inclusive, equitable health system, study participants agreed that some form of HEE should always be considered in CPG decisions, with complex economic analyses reserved for new or expensive technologies. The COVID-19 pandemic has illuminated the need for healthcare decisions to be informed by evidence of effectiveness, feasibility and costs. However, without agreed upon methods or processes for using HEE for decisions, this approach will remain challenging for those who set the CPGs. Presenting HEE in accessible ways for CPG groups is also essential and was flagged as a concern. Addressing these key issues is an imperative precursor to successful cost-informed CPG decision-making in South Africa.

## Supplementary Information

**Additional file 1.** HEE in CPGs consolidated survey results presented by survey question - Dec2020. (XLS 173 kb)

## Data Availability

The dataset supporting the conclusions of this article is included within the article (and its additional file).

## Abbreviations

ATS: American Thoracic Society
CBA: Cost-benefit analysis
CPG: Clinical Practice Guideline
FGP: Focus Group Participants
GRADE: Grading of Recommendations Assessment, Development and Evaluation
HEE: Health Economic Evidence
HITAP: Health Intervention and Technology Assessment Program
iDSI: International Decision Support Initiative
NHI: National Health Insurance
NICE: National Institute for Health and Care Excellence
SAGE: South African Guidelines Excellence
SAMRC: South African Medical Research Council
UHC: Universal Health Coverage
WHO: World Health Organisation
